# Direction-specific enhanced diffusion of CO_2_ in chiral hexagonal boron nitride nanotubes

**DOI:** 10.1038/s41467-026-72123-2

**Published:** 2026-05-28

**Authors:** Manh-Thuong Nguyen, David J. Heldebrant, Jian Liu, Abhoyjit S. Bhown, Zhijie Xu

**Affiliations:** 1 Pacific Northwest National Laboratory, Richland, WA USA; 2 Washington State University, Pullman, WA USA; 3 Electric Power Research Institute, Palo Alto, CA USA

**Keywords:** Computational nanotechnology, Carbon capture and storage

## Abstract

To meet performance requirements, the next generation of gas separation membranes will need *both* high gas permeability and selectivity, attainable if we could coax adsorbates to minimize random Brownian motion and produce direction-specific diffusion along a desired axis. In this atomistic modeling study, we detail how direction-specific diffusion of CO_2_ can be achieved in chiral hexagonal boron nitride nanotubes (hBNNTs) by means of a non-Knudsen diffusion mechanism. Our findings detail how this mechanism of diffusion is driven by interactions with the tube walls and enables the CO_2_ molecules to diffuse along the nanotube’s z-axis with minimized collisions and directional changes. hBNNTs with chiral indices exhibit CO_2_ diffusion rates faster than non-chiral tubes of comparable and larger diameters. Of the hBNNTs studied, a (7,3) tube appears to be ideally sized (3.7 Å radius) exhibiting CO_2_ diffusion that is 3.4 times faster than diatomic N_2_. Applying this mechanism of diffusion to hypothetical sheet membranes prepared with aligned chiral (7,3) hBNNTs results in membranes with a calculated CO_2_/N_2_ permselectivity of 170 and a CO_2_ permeability limit of nearly 1.35 ×10^7^ Barrer, readily surpassing the Robeson upper bound for CO_2_/N_2_ separations.

## Abstract (web summary)

Near perfect direction-specific diffusion of CO_2_ can be achieved in chiral hexagonal boron nitride nanotubes, exhibiting over 3 times faster diffusion than N_2_. Hypothetical sheet membranes using aligned tubes project a CO_2_/N_2_ permselectivity of 170 and a CO_2_ permeability that readily surpasses the Robeson upper bound.

## Introduction

Rotation is a common, omnipresent phenomenon that spans from massive neutron stars to spin in a magnetic field. Rotation can either enhance^1^ or hinder^2^ the transport of an object, depending on how the object interacts with its surrounding medium and other factors. Precession is a special, compound form of rotation that occurs when a spinning object experiences a torque that causes its axis to change direction in a slow, circular motion. Engineering recognizes various observable manifestations of precession, such as the stabilized trajectory of a spinning projectile and the resonance frequency shift in magnetic resonance imaging. Intriguingly, researchers have observed strong mass transport enhancement from miniscule precession^3^, indicating its applicability as a potential strategy for controlling transport processes. Although introducing precession to objects has led to many advancements, its application to molecules in chemical processes has been limited. This could be because linearly symmetrical molecules like CO_2_ cannot “rotate” about their own axis—molecular precession would only be possible if the molecules become slightly bent.

The motion of molecules is key to the gas-phase separations identified as potentially world-changing and important to industries^4–7^. Separations are one of the most common and energy-intensive chemical processes, often using specially designed adsorbents or membranes. Developing next-generation materials and processes for reducing the energy demand and costs for gas-phase separations requires innovative approaches to overcome conventional molecular phenomena. In traditional adsorbents, gases like CO_2_ move via Brownian motion, a chaotic process where molecules flip, tumble, and bounce off interfaces or other molecules at random. Each potential change in direction makes the separation slower and less efficient than if all the molecules moved directionally through a sorbent or membrane.

We posited that we could achieve more efficient separations if we could prevent tumbling and collisions by coaxing molecules into a precession-like motion. Such movement could keep CO_2_ oriented linearly along the pore axis instead of other directions that would increase interactions with the wall and impede axial diffusion. Physical sorbents or membranes with well-defined straight pore channels are preferred targets for imparting molecular precession due to their finely tunable molecular geometries and lack of chemical fixation that would allow CO_2_ to freely rotate inside.

**Fig. 1:**
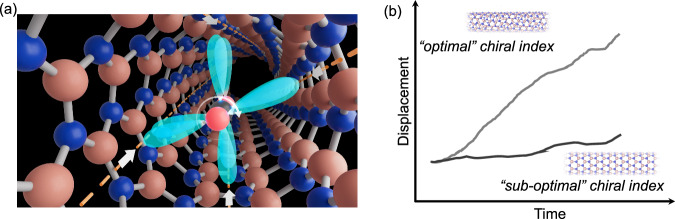
The inspiration for this study. **a** CO_2_ precessing down the z-axis of a chiral hBNNT to avoid collisions. **b** Scheme shows what was discovered: enhanced transport can be achieved via tunable interactions between the substrate and the geometric orientation of hexagonal patterns in the wall of nanotubes. Colour code: B in pink and N in blue.

To design a framework that could introduce molecular precession, we took inspiration from systems where the geometric orientation of a host enhances diffusion and directionality in a transient item. We posited that molecules of CO_2_ could be coaxed into precessing down the horizontal axis of an appropriately sized chiral single-walled nanotube (Fig. 1a). If successful, this would minimize collisions by maintaining the molecule’s orientation along the pore axis and enable rapid, directional diffusion along the *Z*-axis of the nanotube.

Promoting molecular precession requires two primary design criteria: a dipole and an external field. As CO_2_ is a linear quadrupolar molecule, its ${D}_{\infty h}$ symmetry must first be broken to create a dipole that can “precess” in response to an external electric field. There have been reports of CO_2_ molecules being deformed/distorted within nanometer-sized cavities^8,9^, resulting in two π bonding orbitals rotated 90˚ from each other that can act as “fins” to be manipulated via electron repulsion to introduce precession-like motion (Fig. 1a). To satisfy the second criterion, we need to create a continuous rotating electric field for the dipole of the distorted CO_2_ molecule to interact with. hBNNTs can be made with chiral indices and electron-rich nitrogen atoms (Fig. 1a, blue) that create a continuous weak electric field that follows the chiral index of the tube. This field could interact with CO_2_ via electron repulsion to potentially induce precession-like motion. We thus chose to computationally study hBNNT tubes as they meet both of our design requirements: they can break the symmetry of CO_2_ and present a continuous rotating electric field that could potentially coax the distorted molecule into motion that minimizes collisions and directional changes (Fig. 1a). What we found is the first ever evidence that diffusion rates are directly influenced by orientation of hexagonal patterns of boron nitride nanotubes (Fig. 1b).

Here, we detail a machine learning interatomic potential molecular dynamics (MLIPMD) study that describes a type of active motion of CO_2_, representing, to our knowledge, the first-ever direction-specific diffusion of CO_2_ down a desired axis of a sorbent. We will show that this motion can be achieved via coupled interactions of CO_2_ with specific orientations of the hexagonal patterns of the walls of boron nitride nanotubes. Our findings suggest that we can tune these molecular-level interactions via the tube’s chiral indices to minimize the collisions that determine the mean free path of diffusion to govern macroscopic transport. This fundamental phenomenon can occur for CO_2_ (and likely other molecules) under nanoconfinement inside chiral nanotubes, enabling amplified rates of diffusion over non-chiral tubes of similar dimensions while retaining high selectivity. We close with a discussion of how these principles could be introduced to membranes and porous materials to design advanced materials that could exceed or shift the Robeson upper bound for membrane-based CO_2_/N_2_ separations.

## Results and discussion

### Sizing the optimal tube radius for CO_2_ adsorption

**Fig. 2:**
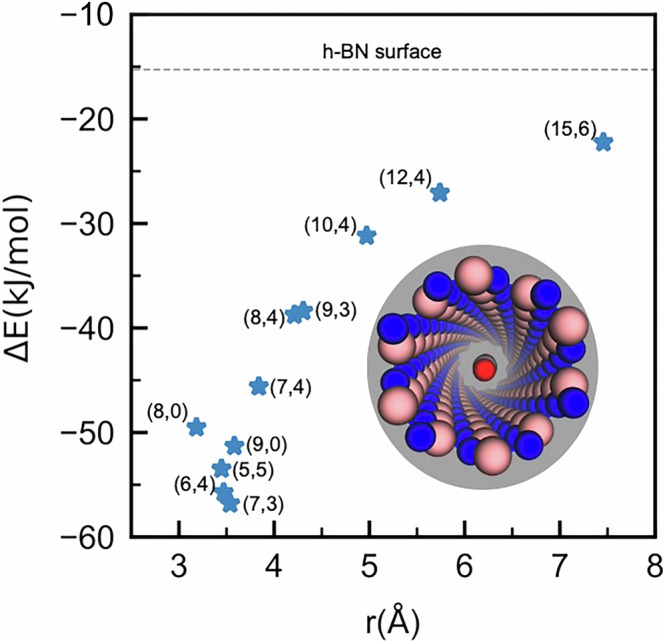
Adsorption energy of CO_2_ in hBNNTs. Colour code: B in pink, N in blue, O in red, and C in grey.

We selected single-walled hBNNTs with commonly made geometries, including armchair, zigzag, and chiral tubes^10^. We first screened the hBNNTs to find optimal dimensions by identifying tubes with a favorable calculated CO_2_ binding energy, Fig. 2. The tube radius, from 3.18 Å ((8,0)) to 7.46 Å ((15,6)), Fig. S1, offers a range of CO_2_–tube interaction scenarios. The binding energy of CO_2_ on a flat h-BN sheet is calculated to be −15.3 kJ/mol, see “Methods” and Fig. S1. This weak interaction, consistent with the literature^11^, indicates that the van der Waals interactions play a key role in the adsorption strength of CO_2_ on the h-BN surface. The binding energy increases for nanotubes relative to a flat surface due to curvature, which raises the density of B and N atoms surrounding the CO_2_. This becomes more obvious upon further decreasing the tube radius. Of the tubes under study (indices provided in Fig. 2), the (7,3) tube interacts most strongly with CO_2_, with a binding energy of −57 kJ/mol (“−” implies attraction). The radius of this tube, 3.54 Å, is near the equilibrium distance of CO_2_ on the h-BN surface, 3.55 Å. CO_2_ is very close to the wall in tubes with smaller radii, resulting in Pauli repulsions and weakening of the CO_2_-hBNNT bonding.

### Molecular diffusion in selected tubes

**Fig. 3:**
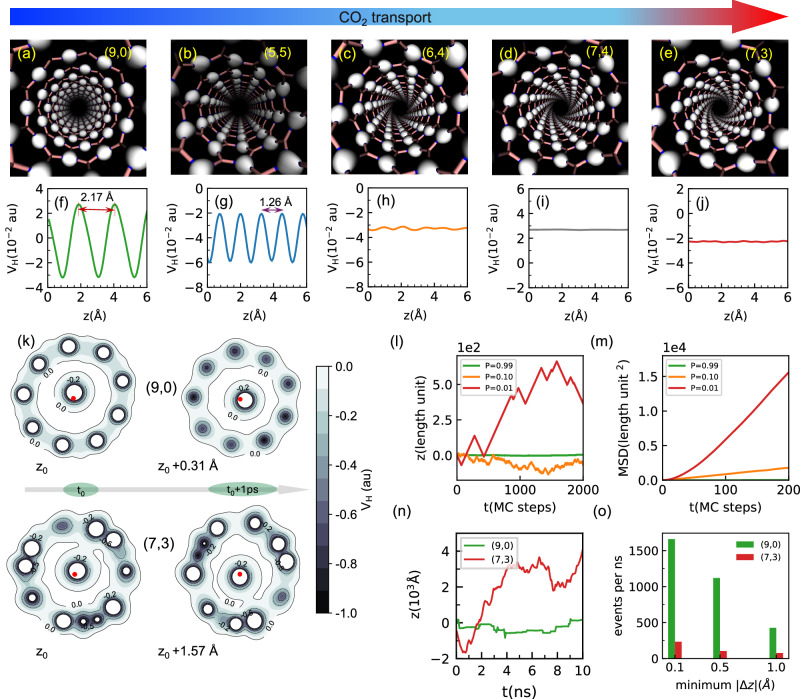
Transport of CO_2_ is connected to electronic properties of hBNNTs. **a**–**e** Visualization of electron clouds (density, iso-surface value = 0.3 au); **f**-**j** corresponding xy-average electrostatic potential VH (within 3.0 Å of the tube center, see S. 1.3 for more details)along the tube direction; **k** cross-section of V_H_ at the position of atom C (of CO_2_) at 2 representative snapshots (1 ps apart) for the (9,0) and (7,3) systems, *t*_0_ denotes the position of C at a time *t*_0_ and the red dot denotes the tube’s center; **l** the position of the walker in 1-dimensional MC simulations versus time (number of MC steps) with P being the probability of changing direction (left vs right); and **m** the corresponding MSD versus time (averaged from 1000 independent runs); **n** position of CO_2_ in the tube vs time from MD; and **o** number of events that CO_2_ reverses its moving direction (along the z direction), per ns, after travelling a minimum distance |Δz|.

Having identified tubes with large binding energies, we focused on probing CO_2_ diffusion in tubes with similar radii, but different orientations of the hexagonal patterns. We selected one armchair (5,5), one zigzag (9,0), and three chiral tubes [(6,4), (7,3), and (7,4)] with differing degrees of twist and 3.45, 3.58, 3.47, 3.54, and 3.84 Å radii, respectively (the larger (7,4) tube was selected for comparison). These differences result in varied fundamental electronic properties, such as the distribution of electron clouds (Fig. 3a–e) or the xy-averaged electrostatic potentials inside the tubes (Figs. 3f–j and S2). Additionally, the selected tubes exhibit the highest N_2_ adsorption capacities among those investigated (Fig. S3). Separations of CO_2_ and N_2_ using on hBNNTs will be discussed later.

MLIPMD simulations were subsequently conducted to determine the diffusivity of CO_2_. We calculated the self-diffusion coefficient along the tube axis direction (*D*_*self*_) using totally 10 ns statistics for each system, see “Methods” and Fig. S4. *D*_*self*_ of CO_2_ in the hBNNTs, Table 1, shows the tubes in increasing order of diffusivity: (9,0) << (5,5) < (6,4)  ≤ (7,4) << (7,3), indicating that *D*_*self*_ does not necessarily anticorrelate with the binding energy, see Fig. S5. It should be noted that the calculated *D*_*self*_ from these simulations are for CO_2_ with a flexible (slightly bent, for example, 174.1˚ in (7,3) on average) geometry that in part arises from interactions with the nitrogen orbitals inside the tubes. If CO_2_ was constrained in a rigid linear geometry in a (7,3) tube, *D*_*self*_ would drop by more than 2 times. This is consistent with previous studies that have shown faster diffusion for distorted, or “flexible” CO_2_ molecules over linear, rigid ones^12^. Under confinement, deviations from linearity can reduce steric repulsion with the pore wall and enhance reorientational/librational motion, thereby strengthening translation-rotation coupling and facilitating transport. It is necessary to point out that the intrinsic bending mode of CO₂ lies at approximately 667 cm^−1^ and remains largely quantum mechanical at room temperature, with its amplitude dominated by the ground-state wavefunction. Accordingly, numerical similarity between classical and quantum vibrational amplitudes is not interpreted as evidence that the bending dynamics are accurately described classically. In a confined pore, however, symmetry breaking leads to mixing between translational, rotational, and vibrational degrees of freedom, giving rise to low-frequency, thermally populated motions with a partial bending character. These nanoconfinement-induced modes are sensitive to the external potential and are well described classically. They can indirectly modulate the effective bending geometry and orientation of CO₂ without requiring direct classical treatment of the high-frequency quantum bending vibration.

**Table 1:** Calculated self-diffusivity (*D*_*self*_), Knudsen diffusivity (*D*_*K*_), and selectivity in chiral and non-chiral nanotubes

Tube	Chiral	Tube diameter(Å)	CO_2_			N_2_			CO_2_/N_2_
			*D*_*self*_ (10^−9^ m^2^/s)	*D*_*K*_(10^−9^ m^2^/s)	*D*_*self*_*/D*_*K*_	*D*_*self*_ (10^−9^ m^2^/s)	*D*_*K*_(10^−9^ m^2^/s)	*D*_*self*_*/D*_*K*_	*D*_*self*_
(9, 0)	No	7.16	271 (± 128)	90	3	1017 (± 135)	113	9	0.27
(5, 5)	No	6.90	2814 (± 299)	87	32	2077 (± 348)	109	19	1.35
(6, 4)	Yes	6.94	3252 (± 256)	88	37	2402 (± 326)	110	22	1.35
(7, 4)	Yes	7.68	3266 (± 268)	97	34	1666 (± 203)	122	14	1.96
(7, 3)	Yes	7.08	5521 (± 305)	89	62	1625 (± 228)	112	15	3.40
(7, 3)_rigid_	Yes	7.08	2443 (± 151)	89	28				

In the case of (7,3)_rigid_, CO_2_ was constrained in a rigid linear geometry.

Interestingly, there was no clear correlation between *r* and *D*_*Self*_, contradicting the notion that diffusion is faster in smaller diameter tubes^13,14^. The (5,5) and (6,4) tubes have very similar radii (0.6% difference), though the latter exhibits a ~ 1.2-fold enhancement in transport efficiency. Similarly, (9,0) has a 1.1% larger radius than (7,3); however, the CO_2_ diffusion in (9,0) is 20 times slower. At first glance, the significantly slower diffusion of CO_2_ in (9,0) is aligned with distinct peaks in the radial distribution functions of the C-B or C-N atom pairs (Fig. S6), different from the single peak feature of other studied systems. Nevertheless, the faster diffusion in the chiral tubes (particularly, in (7,3)) and lower diffusion in non-chiral tubes are attributed to various factors.

Of these factors, electrostatic potential is particularly important in our system due to the nature of the CO_2_-hBN interactions (Van der Waals), with the Pauli repulsion between electron-rich atoms (O of CO_2_ and N of hBN) heavily emphasized. The O atoms of CO_2_ are likely to be at any locations inside the tube where repulsion is minimized. As such, the “localization” of CO_2_ along the z-direction hinders the molecule’s transport. Figure S7a–e shows the potential of mean force (PMF) with the z-component of the O-N distance as the reaction coordinate. The O atoms, and thus the CO_2_ molecule, clearly favor particular locations in (9,0): the distance between two such locations along the z-direction is the same as the distance between two peaks of the electrostatic potential (Figs. S7a and 3f). To a lesser extent, CO_2_ shows the same behavior in (5,5) (Figs. S7b and 3g). In the chiral tubes, the distribution is mostly even, aligning with the flat electrostatic potentials (Figs. S7c–e and 3h–j). The electrostatic maps in Fig. 3k demonstrate the rotation of CO_2_ about the tube axis. They also show high and low symmetry of the tube’s electrostatic potential within a plane for (9,0) and (7,3), respectively. In contrast, the (average) potential along the z direction, which is subject to rotation, varies more significantly in (9,0) than in (7,3), which is consistent with the xy-average potentials shown in Fig. 3f, j). The electron-rich O atoms of CO_2_ can experience stronger external local fields in non-chiral tubes than in chiral tubes due to the roughness of the electrostatic potential along the z-direction, Fig. 3f–j, decreasing CO_2_ diffusivity through the non-chiral tubes.

How the roughness of the electrostatic potential hinders the diffusion of CO_2_ along the z-direction of tubes can be further interpreted through Monte Carlo (MC) simulations of a 1-dimentionsal walker. Figure 3l–m demonstrates how the walker’s (CO_2_’s) mobility depends on the probability of reversing its moving direction. Higher reversal probabilities constrain the walker to oscillate around its initial position, yielding minimal net displacement. As the probability decreases, directional persistence increases, allowing the walker to move progressively farther from its origin. This transition forward, i.e., more extended diffusion, is captured by the corresponding rise in MSD, indicating that lower probabilities promote enhanced diffusivity. The MC results mirror the diffusion of CO_2_ in the (9,0) and (7,3) tubes. Figure 3n-o shows that CO_2_ moves much further in (7,3) than in (9,0) as time elapses and that CO_2_ reverses its direction after travelling a given minimum distance much more frequently in (9,0) than in (7,3). The motion of CO_2_ within the tube, including rotational behavior, may directly impact the collisions between the molecule and the walls.

### Precession-like behavior of CO_2_

**Fig. 4:**
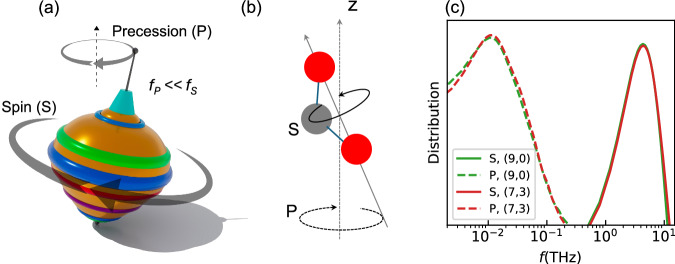
Precession and Rotational Dynamics of CO₂ in hBNNTs. **a** Precession of a spinning top; **b** scheme of two different rotations of CO_2_, with C in grey and O in red; **c** frequency of rotation of CO_2_ in hBNNTs.

Consistent with our initial hypothesis for this work, the CO_2_ molecules were found to precess inside the tubes. Figure 4a shows the precession of a spinning top, with its two different co-occurring rotations: (1) spin, or the fast rotation about its own axis (S), and (2) the slow rotation of the spin axis about an external axis (P). Linear CO_2_ is less diffusive than flexible CO_2_, suggesting that molecular rotation may play an important role in this process. In this section, we analyze the rotation of CO_2_ (specifically the C atom) about its own OO axis, facilitated by its bent geometry (Fig. 4b), and the rotation of the OO axis about the tube axis direction ($\vec{z}$), facilitated by the tilting of OO with respect to ($\vec{z}$) (Fig. S8). Here we focus on the two extreme cases: (9,0) and (7,3) tubes.

Movies S1–4 highlights both the slow, conics-like motion of the OO axis about the tube direction and the fast rotation of the molecular plane. The calculated S and P rotation frequency spectra (see Section S1.5 for additional details) are shown in Fig. 4c. In the two tubes, what we define to be a precession frequency is much lower (about two orders of magnitude) than the spin frequency, making it similar to the extremely slow precession trend of well-known objects such as Earth. To further understand the precession-like behavior of CO_2_, we calculated the torque of a rigid, bent molecule in the two tubes (Fig. S9). While this artificial geometry is not physically accurate, it can be useful for interpreting the precession and the role of molecule-tube interactions in the absence of internal degrees of freedom. The z component of the torque (${\tau }_{z}$), which makes the molecule rotate about the z direction, is higher in the (7,3) than in (9,0)—consistent with the trend of ${f}_{P}$ in these tubes. While the precession in (9,0) may not help CO_2_ reduce collisions with the tube wall, Fig. 3k, the asymmetry of the potential may help in the (7,3) tube. Note that there are several factors dictating the unique transport of CO_2_, we are pursuing further research into the underlying physics of the observed phenomena and will present it in a follow-on publication.

### Overcoming Knudsen diffusion and comparison with N_2_ transport

Better understanding the effects of the topology of the interior of the chiral hBNNTs requires comparison with conventional diffusion mechanisms. We thus consider the Knudsen mechanism for both CO_2_ and N_2_ as a benchmark to determine if the precession-like motion has a significant effect on diffusion. Knudsen diffusion is active in our chosen system as the pore diameters (< 8 Å) of the studied hBNNTs are much smaller than the mean free path of both CO_2_ and N_2_ gas molecules and the densities of the gases are low, meaning that the gas molecules will collide with the pore walls more frequently than with other molecules^15^. The Knudsen diffusivity ${D}_{K}$ was calculated as

1 $${D}_{K}=\frac{d}{3}\sqrt{\frac{8{RT}}{\pi M}}$$

where $d$ is the pore diameter (m), $R$ is the gas constant (J/(mol K)), $T$ is the temperature (K), and $M$ is the molar mass (kg/mol)^16^. The ${D}_{K}$ of CO_2_ and N_2_ in different nanotubes were calculated and compared with the *D*_*self*_ (shown in Fig. 3) in Table 1.

In the (9,0) tube, the *D*_*self*_ of CO_2_ is closer to the *D*_*K*_. Distinctly different from this, in the chiral hBNNTs, the *D*_*self*_ of CO_2_ is much larger than the *D*_*K*_, indicating a deviation from conventional diffusion mechanisms at these scales. The large ${D}_{{self}}/{D}_{K}$ ratio is particularly encouraging as the CO_2_/N_2_ Knudsen selectivity of 0.8 indicates that CO_2_ being larger should exhibit inherently slower diffusion compared to N_2_. Note that the CO_2_ ${D}_{{self}}/{D}_{K}$ ratio in (5,5) is slightly reduced compared to (6,4) or (7,4), potentially due to the hyperloop effects discussed later.

Knudsen diffusion depends primarily on the pore size and the molecular mass (Eq. (1)), ignoring the rotational/vibrational degrees of freedom of CO_2_ and interactions with the tubes. The amplification of self-diffusion over Knudsen diffusion, indicative of enhanced CO_2_ diffusion in the chiral tubes, may be attributed to rotational degrees of freedom minimizing molecular collisions with the tube walls (see Supplementary Movies S1–4). The enhanced diffusivity of flexible CO_2_ compared to linear CO_2_, Table 1, demonstrates that the motion of a slightly bent CO_2_ about its O–O axis (impossible in the rigid, linear geometry) significantly contributes to the molecule’s ability to precess through the tubes, minimizing collisions and directional changes, resulting in enhanced transport. In the non-chiral tubes, CO_2_ still precesses, but experiences rougher free energy surfaces. This difference is reflected in the PMF and electrostatic potentials, Fig. S7 and Fig. 3f–j, resulting in more regular directional reversals and slower diffusion.

The relatively high CO_2_ ${D}_{{self}}/{D}_{K}$ in non-chiral (5,5) can be linked to a reported “hyperloop” effect, which has shown that molecules confined and aligned within a 1-dimensional nano channel exhibit greatly enhanced diffusion^14^. Since this effect emerges when the molecular axis aligns with the nanochannel axis, we monitored the orientation of CO_2_ within the tubes. The degree of alignment between CO_2_ and each tube is quantified by α, defined as the angle between the O–O molecular axis and the tube axis (Fig. S9); smaller α values indicate greater alignment with the channel. The average value (in ^o^) of *α* is 11.6, 9.0, 9.6, 17.9, 10.7, and the full width at half maximum (in ^o^) of its distribution is 14.7, 11.3, 12.2, 22.0, and 13.5, in the case of (9,0), (5,5), (6,4), (7,4), and (7,3) tubes, respectively. In comparison to CO_2_ in chiral (6,4), CO_2_ in non-chiral (5,5) exhibits slightly more enhanced alignment and smaller variation in O–O direction, suggesting a lower degree of tumbling and a higher degree of hyperloop enhancement. This would counterbalance the stronger collisions between the molecules and nitrogen electron clouds in (5,5), leading to a similar ${D}_{{self}}/{D}_{K}$ ratio to (6,4), Table 1. N_2_, however, exhibits very different behavior. In all tubes, N_2_ undergoes tumbling during diffusion, retarding transport. In smaller tubes (5,5) and (6,4), N_2_ exhibits higher diffusivity due to less frequent tumbling.

### Influence of the mechanics of tube geometry and wall interactions

To further our understanding of chirality-enhanced molecular transport for different molecules, we employed mathematical modeling that incorporates molecule-wall interactions that vary with molecular properties. As Knudsen diffusion depends primarily on the tube diameter and molecular mass (Eq. (1)), it neglects any mechanical interactions between the molecules and the tube walls. As the nanotube diameter approaches the molecular scale (~ 3.3 Å for CO_2_), wall roughness and atomic-scale forces become significant, making the point-like particles approximation for the gas molecules invalid. In this scenario, the molecule-wall interactions play a more prominent role.

**Fig. 5:**
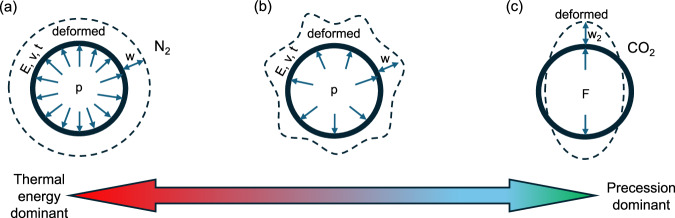
Schematic plot of different modes of molecule-wall interaction for N_2_ and CO_2_. **a** Thermal energy is dominant for N_2_ molecules. As diatomic N_2_ does not exhibit molecular precession, the random nature of thermal noise tends to lead to a uniform molecule-wall interaction and deformation w; **b** an intermediate case between the two extremes; **c** molecular precession dominates over thermal energy for CO_2_ interacting with nanotubes with strong chirality. The molecule-wall interaction tends to be less uniform and more concentrated, leading to a larger, localized deformation w_2_ due to the effect of stress concentration.

Figure 5 provides a qualitative description of the effect of precession on the molecule-wall interaction for different gas molecules where, *E*, *v*, and *t* represent the circumferential Young’s modulus, Poisson ratio, and tube thickness. At present, the causality between the molecule-wall interactions and precession-like molecular motion is unclear, but competition between thermal energy and molecular precession could lead to different modes of molecule-wall interactions and a different diffusion mechanism. Regardless of the specific underlying phenomena, we posit that the motion of CO_2_ molecules is coupled to interactions with the wall along the chiral index, while that of N_2_ is likely to be negligible.

For diatomic N_2_ molecules, there is no molecular precession imparted by the tube wall. Thermal energy is dominant, leading to a more uniform molecule-wall interaction mode due to the random nature of thermal noise. The corresponding wall deformation *w* is relatively uniform and small along the tube circumference. To compare the two deformations, we can calculate the uniform deformation *w* subject to a uniform load *p*:^17^

2 $$w=\frac{p}{2E}{[3(1-{v}^{2})]}^{\frac{1}{4}}{\left(\frac{R}{t}\right)}^{\frac{3}{2}}$$

where *R* and *t* are the tube size and thickness.

At the other extreme, the transport mechanism imparted by wall interactions along the chiral index with CO_2_ molecules could be strong enough to dominate over thermal energy. In this scenario, the coupled molecule-wall interactions would be more concentrated and the corresponding wall deformation w_2_ would localize and become much larger than the uniform deformation *w* due to the effect of stress concentration. For comparison, we also calculate the localized deformation *w*_*2*_ under concentrated load *F*^18^

3 $${w}_{2}=\alpha \frac{3\sqrt{2}(1-{v}^{2})}{\pi }\frac{F}{Et}{\left(\frac{R}{t}\right)}^{2}$$

where α is a numerical factor of order unity. For a fair comparison, the two loads should be related as *F* = *2pR*, as a result of requiring the same total molecule-wall interaction. With the Poisson ratio *ν* = 0.16261^19^, the rough estimate of the ratio between the two deformations now becomes: *w*_2_/*w* ≈ *4(R/t)*^*3/2*^, dependent on the ratio *R/t*. For single-wall hBNNTs, the size of the nanotube is comparable to the thickness^20^ and the maximum ratio of localized to uniform deformation is around 4. This much larger localized deformation *w*_2_ may contribute to the faster gas transport of CO_2_ in chiral tubes. In contrast, smaller and uniform deformation may hinder the N_2_ transport. This is directly analogous to driving a screw into wood, where the sharp threads concentrate stress and cause larger localized deformation, displacing material to enable fast forward motion along the axis.

The same mechanism is unlikely to operate for N_2_ as it is a linear diatomic molecule that cannot bend and undergo precession-like motion like CO_2_. Therefore, thermal energy always dominates for N_2_ molecules, leading to uniform molecule-wall interactions. Thus, there is no localized deformation in chiral tubes for N_2_ molecules, consistent with calculated diffusion rates for N_2_ being slower than CO_2_ as described above. While probing the specific underlying physical phenomena lies beyond the scope of this work, follow on studies of the physics and mechanics of the molecule-wall coupled interactions will shed light onto the observed enhanced diffusion.

### Studying diffusion for membrane applications

We assessed the potential performance of a hypothetical CO_2_-separating membrane (most dense packing of an array of aligned cylindrical nanotube) with the selectivity and direction-specific diffusion found in the chiral BNNTs. It has been well documented that there is often a tradeoff between permeability and selectivity for polymer membranes. The relationships for many gas pairs were summarized into a series of plots known as the Robeson upper bound^21^. These upper-bound results were updated for polymer membranes in 2008 and are commonly used as a reference to understand the performance of gas separation membranes^22^. The transport of gases in a hypothetical membrane made of chiral (and non-chiral) hBNNT assembly can be described using a solution-diffusion mechanism^23^

4 $${J}_{{CO}2}=\left(\frac{{D}_{{CO}2}\varepsilon }{\tau }\right){A}_{m}{S}_{{CO}2}\frac{{\triangle f}_{{CO}2}}{l}$$

where the *J*_CO2_ is the total flux of CO_2_ across the membrane, *D*_CO2_ is the CO_2_ diffusion coefficient in the nanotube, *ε* is the membrane surface porosity, *τ* is the membrane pore tortuosity, *A*_m_ is the membrane surface area, S_CO2_ is the solubility coefficient of CO_2_ defined as the ratio of CO_2_ concentration in the nanotube to the CO_2_ fugacity in the gas phase, Δ*f*_CO2_ is the fugacity difference between the bulk gas phases on either side of the membrane, and *l* is the membrane thickness. Based on this mechanism, the permeability coefficient of the membrane to CO_2,_ *P*_CO2,_ can be written as

5 $${P}_{{CO}2}={S}_{{CO}2}\times {D}_{{eff},\,{CO}2}$$

where *D*_*eff*_,_CO2_ is the term in parenthesis in equation 4, called the effective diffusion (transport) coefficient of CO_2_^24^. The effective diffusion coefficient was calculated based on the self-diffusion coefficients for the hBNNTs calculated in the molecular dynamics simulations detailed above (Table 1) multiplied by the porosity of the membrane and divided by the tortuosity of the pore in a membrane.

For an upper estimate of the effective diffusion coefficient, we assumed that a hypothetical membrane is made from a close-packed array of aligned cylindrical nanotubes, resulting in an average membrane porosity of 0.9069 and a pore tortuosity of 1. These self-diffusion coefficients of CO_2_ in the BNNTs are comparable to those calculated for CO_2_ diffusion in a (10,10) single-wall carbon nanotube at similar pressures with CO_2_^25^. These self-diffusion coefficients are often used as a conservative approximation for the transport diffusion coefficient, reported for calculating the diffusion of argon (Ar) in carbon nanotubes ^26^.

For the (7,3) hBNNT, the effective diffusion coefficient ${D}_{{eff},{CO}2}=5521\times \left(\frac{\varepsilon }{\tau }\right)=5521\times 0.9069=5007\,$(10^−9^ m^2^/s) for the ideal membrane represents this upper limit. The porosity of some practical membranes range from 0.15 to 0.5^27^. A common relationship between the porosity and tortuosity of porous media is described by the Bruggeman equation^28^

6 $$\tau={\varepsilon }^{-\frac{1}{2}}$$

The corresponding tortuosity can be estimated from 2.6 to 1.4. A possible lower limit of the effective diffusion coefficient of CO_2_ in a practical membrane is calculated to be ${D}_{{eff},{CO}2}=5521\times \left(\frac{\epsilon }{\tau }\right)=5521\times 0.0577=319$ (10^−9^ m^2^/s) using the same method shown above. Therefore, a possible lower limit of membrane permeance is about 1/15 that of the ideal membrane composed of the aligned array of nanotubes.

The CO_2_ solubility coefficients for the hBNNTs were calculated by estimating the change in the Gibbs free energy from the bulk gas phase to inside the membrane pore. For simplicity, the calculation was done at room temperature and 1-atm pressure, where $\triangle G=\triangle {G}^{0}$ (see section S1): −2.0 kJ/mol (in (7,3) tube) for CO_2_ and +7.7 kJ/mol (7,3) for N_2_. From this, the above-defined solubility coefficient was calculated using the relationship between Henry’s law constant and the change of standard chemical potential (equivalent to Gibbs free energy per mole)^29^

7 $$S=\frac{1}{{RT}}\exp \left(-\frac{\triangle {G}_{0}}{{RT}}\right)$$

8 $$\triangle G=\triangle {G}_{0}-{RT}{{\mathrm{ln}}}(f/{f}_{0})$$

where $\triangle G$ is the Gibbs free energy at fugacity of $f$, and ${f}_{0}$ is the fugacity at standard condition 1 atm, *R* is the gas constant and *T* is the temperature. Thus, the pure-component solubility CO_2_/N_2_ selectivity for (7,3) hBNNT is approximately 50, and the pure-component diffusion selectivity is 3.40 for CO_2_/N_2_. When combined, the overall pure-component permeability-selectivity for CO_2_/N_2_ in (7,3) hBNNT is estimated to be 170. This high permeability-selectivity suggests that hBNNT can be used for CO_2_/N_2_ separations. For the (7,3) hBNNT, the CO_2_ solubility coefficient was estimated to be 9.17 $\times$ 10^−2^ mol/L-atm while the N_2_ solubility coefficient was estimated to be 1.83 $\times$ 10^−3^ mol/L-atm. The permeance of the ideal aligned nanotube membrane can be calculated by multiplying the effective diffusion coefficient (5007 $\times$ 10^−9^ m^2^/s) and solubility coefficient (9.17 $\times$ 10^−2^ mol/L-atm). After converting the unit of permeability to a common unit, Barrer (10^−10^ cm^3^(STP) cm^2^ cm^−3^ cmHg^−1^ s^−1^), the permeability of the ideal membrane was estimated to be about 1.35 $\times$ 10^7^ Barrer, considered as the upper limit. A potential lower limit of the permeance, as previously mentioned, was estimated to be about 8.61 $\times$ 10^5^ Barrer.

**Fig. 6:**
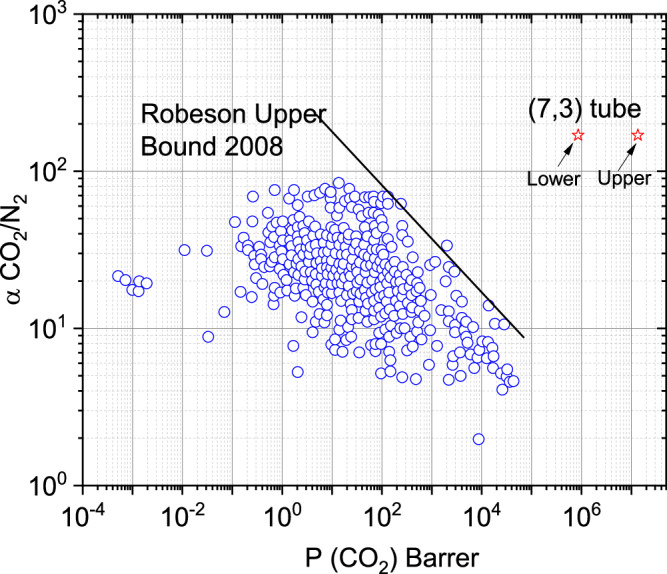
The calculated CO_2_ permeability coefficient in the 2008 Robeson upper bound relationship, data taken from ref. ^22^. The star symbols define the possible lower and upper limits of the calculated permeability coefficients for the (7,3) hBNNTs studied here.

Combined, the permeability-selectivity and upper and lower limits of the permeability coefficients for membranes made with the upper and lower limits of (7,3) hBNNTs were added to the 2008 Robeson upper bound plot of CO_2_/N_2_ separation (Fig. 6). These hypothetical membranes made of hBNNTs have the potential to break the 2008 upper bound for CO_2_/N_2_ separation. Even when the nanotubes (7,3) were included in a practical polymer matrix with the porosity and tortuosity described above, the permeance and the CO_2_/N_2_ ratio of that membrane still would make it above the upper bound. Our results suggest that direction-specific diffusion could be a property that enables the design of next-generation gas separation membranes with *both* high gas permeability and selectivity. We are currently extending this work to include the concentration dependence of diffusivity, solubility, and permeability, as well as gas mixtures of CO_2_ and N_2_ instead of CO_2_ and N_2_ as pure components. These calculations will be useful to help guide potential separation applications.

### Expansion outside of separations of CO_2_

This mechanism of diffusion may not be unique to CO_2_ and may have been undetected for other molecules under nanoconfinement in chiral nanotubes. The literature includes a wealth of papers detailing the unexpectedly high diffusion and transport of water through some small-diameter CNTs. Holt et al. reported water flow rates that exceeded predictions from the Knudsen diffusion model by more than an order of magnitude and flow that exceeded values calculated from continuum hydrodynamics models by more than three orders of magnitude^30^. Tunuguntla et al. discovered highly accelerated water flow in short (∼10-nm) fragments of CNTs embedded in lipid bilayer membranes^31^. While not specifically mentioned, the fast diffusion was observed in chiral CNTs. The modeled water molecules appear to be aligned in a linear fashion with a discernible rotation that matches the chiral indices of the CNT. Cambré et al. showed experimental evidence of water filling a chiral CNT with the molecules aligning single file^32^, moving with a periodicity that appears to map to the CNT’s chiral indices. These two studies suggest that the influence of chirality could impart a previously unconsidered means of diffusion for water (or clusters), similar to our findings on CO_2_. Attempts to find more references about the effects of chiral tubes on water transport were limited as we found no mentions of the chiral indices of simulated CNTs, only diameters. We posit that a more thorough reassessment of the chirality of CNTs in prior work could elucidate whether undetected molecular precession may have influenced the rapid diffusion of water. If true, this finding may shed more light on why some CNTs rapidly move water while others do not. As such, we are currently initiating simulations to study the transport of water and other molecules in chiral nanotubes.

### Managing expectations: limits of experimental approaches

While this work has identified a molecular-level phenomenon with significant promise, we would be remiss if we did not highlight that these findings are from a computational study and not are not yet experimentally validated. A wide range of hBNNTs have already been synthesized, isolated, and characterized. Given sufficient quantities of pure hBNNTs, high-pressure magic angle spinning nuclear magnetic resonance, more specifically diffusion ordered spectroscopy, could measure diffusion coefficients of ^13^C-enriched CO_2_ condensed inside the tubes. This approach has been used to study diffusion coefficients of CH_4_^33,34^, CO_2_^33^, and H_2_O^33^ in nanoporous media. Assessing the chirality of CO_2_ diffusing through the tubes will be far more difficult to experimentally observe. We are in the process of designing such measurements by means of polarized light or polarized neutrons.

## Summary

This computational study presents evidence of a type of coupled transport for CO_2_ confined inside chiral nanotubes that results in faster than Knudsen diffusion. hBNNTs distort CO_2_ by ~5˚ through nanoconfinement and, when the chiral index provides a continuous twisted field, can coax CO_2_ into molecular precession-like motion. Our calculations suggest that the (7,3) tubes are perfectly sized, achieving a high selectivity of CO_2_/N_2_. Chiral hBNNTs exhibit diffusion rates faster than non-chiral tubes of comparable or larger diameters. The (7,3) hBNNT appears optimal, enabling CO_2_ to diffuse faster than N_2_ with a CO_2_/N_2_ diffusion selectivity of 2.06. The predicted performance of a hypothetical sheet membrane prepared with aligned chiral (7,3) hBBNTs shows a CO_2_/N_2_ permselectivity of 170 and a CO_2_ permeance of 1.35 $\times$ 10^7^ Barrer, readily surpassing the Robeson upper bound for CO_2_/N_2_ separation.

If the selectivity and diffusion can be experimentally validated, this molecular-level phenomenon could be further refined or exploited to design more advanced sorbents that could entail amplified diffusion by means of near-perfect direction-specific non-Knudsen diffusion of adsorbates down their axis. It is likely that molecular precession could be imparted by chiral carbon nanotubes or other porous materials like metal-organic frameworks, covalent-organic frameworks, and zeolites. We also assume that this phenomenon is not unique to CO_2_ and that other molecules, like water, could achieve directional-specific diffusion down the axis of an intelligently designed sorbent.

## Methods

### Ab initio molecular dynamics (AIMD)

AIMD simulations were conducted using the CP2K program^35^. We employed the PBE-D3 density functional^36,37^ and the hybrid Gaussian–Plane wave basis set framework^38^, in which the DZVP Gaussian basis functions^39^ in conjunction with a plane wave cut-off of 450 Ry were used. The Goedecker-Teter-Hutter pseudopotentials^40^ with valance states of B(2s^2^ 2p^1^), C(2s^2^ 2p^2^), N(2s^2^ 2p^3^), and O(2s^2^ 2p^4^), were adopted. In self-consistent calculations, only the Γ-point was considered. All molecular dynamics simulations were carried out within the NVT ensemble, in which the temperature was maintained at 300 K with the canonical sampling through velocity rescaling thermostat^41^. The time step was set at 1.0 fs. Each system was equilibrated for 10 ps, followed by a 70-ps production run. For simulations involving rigid CO_2_, the three internal bond distances (C–O, O–C, and O–O) were constrained using the SHAKE algorithm^42^, with a tolerance of 5 $\times$ 10^−5^.

### Static density functional calculations for binding energies

The binding energy of a (confined) CO_2_ or N_2_ molecule (mol) in a hBNNT was calculated as

9 $$\varDelta E=E\left({mol}-{hBNNT}\right)-E\left({mol}\right)-E\left({hBNNT}\right)$$

in which the energy *E* of each system was calculated at the density functional level provided above.

### Deep potential model training

We used the deep potential (DP) model^43^, implemented in the DeePMD-kit^44^, to train machine learning interatomic potentials. Very briefly, the total potential energy of a system is given by

10 $$E={\sum}_{i}{E}_{i}={\sum}_{i}N({D}_{i}({{{{\boldsymbol{R}}}}}_{i}))$$

in which ${{{{\rm{E}}}}}_{{{{\rm{i}}}}}$ is the local atomic energy determined by atom i and its surrounding environment within a cutoff ${R}_{c}$, the symmetry-preserving descriptor ${D}_{i}$ is the feature matrix encoding the surrounding environment and is fed to a deep neural network *N* which returns the energy ${E}_{i}$. ${{{{\boldsymbol{R}}}}}_{i}$ denotes the set coordinates of all atoms in the environment, ${{{{\boldsymbol{R}}}}}_{i}=\left\{{{{{\boldsymbol{r}}}}}_{{ij}}\equiv {{{{\boldsymbol{r}}}}}_{i}-{{{{\boldsymbol{r}}}}}_{j}\right\}.$

The network is trained through the minimization of the loss function

11 $${{{\mathscr{L}}}}={p}_{E}{|\triangle E|}^{2}+\frac{{p}_{f}}{3N}{\sum}_{i}{|\triangle {F}_{i}|}^{2}$$

where $\triangle E$ and $\triangle F$ are the deviation of the potential energy and atomic forces between the reference DFT and predicted data, respectively; and ${p}_{E}$ and ${p}_{f}$ are tunable prefactors.

We used a {20,40,80} embedding and {200,200,200} fitting network. The radial cutoff and the smooth cutoff were set at 6.5 and 4.5 Å, respectively. The prefactor *p*_*E*_ was set to increase from 0.02 to 1 and *p*_*f*_ was set to decrease from 1000 to 1.

From 70,000 AIMD frames, 56,000 frames were randomly chosen to create a training set, similarly, 7000 frames for a validation set and 7000 frames for a test set. The accuracy of trained potentials is provided in Table S1.

### Machine learning interatomic potential molecular dynamics (MLIPMD)

MLIPMD simulations were carried out using LAMMPS^45^. We used the NVT ensemble in which the temperature (300 K) was maintained using the Nose-Hoover thermostat^46^. The time step was set at 1 fs. Five independent simulations were conducted for each system, each comprising a 0.5 ns equilibration followed by a 2.0 ns production run with atomic coordinate recorded every 20 fs. This resulted in a total of 10 ns of production data per system for statistical analysis. In simulations with rigid CO_2_, the “rigid/nvt molecule” command was applied to constrain intramolecular motion while maintaining a constant temperature ensemble.

### Diffusion coefficient

The self-diffusion coefficient of CO_2_ along the tube direction z, *D*_*self*_, was calculated from the mean square displacement (*MSD*_*z*_)

12 $${D}_{{self}}=\frac{1}{2}{{{\mathrm{lim}}}}_{t\to \infty }\frac{d}{{dt}}{{MSD}}_{z}(t)$$

13 $${{MSD}}_{z}\left(t\right)=\left\langle {\left|{r}_{z}\left(t\right)-{r}_{z}\left(0\right)\right|}^{2}\right\rangle$$

in which ${r}_{z}$ is the *z* component of the centroid coordinate of a CO_2_ molecule and the <> notation indicates the ensemble average.

## Supplementary information

Supplementary Information File

Transparent Peer Review file

Description of Additional Supplementary Files

Supplementary Dataset 1

Supplementary Dataset 2

Supplementary Dataset 3

Supplementary Dataset 4

Supplementary Movie 1

Supplementary Movie 2

Supplementary Video 3

Supplementary Video 4

Supplementary Code 1

## Source data

Source Data

## Data Availability

The MLIP files are available upon request. Source data for figures in the main text are provided with this paper. Structures (in the xyz format) of CO_2_ and N_2_ in the (7,3) and (9,0) hBNNTs are provided as files (Supplementary_XYZ_1_CO2_hBN73.xyz, Supplementary_XYZ_2_CO2_hBN90.xyz, Supplementary_XYZ_3_N2_hBN73.xyz, Supplementary_XYZ_4_N2_hBN90.xyz). Source data are provided with this paper.
