# Supplementary material for: Direction-specific enhanced diffusion of CO2 in chiral hexagonal boron nitride nanotubes
Source: Nat Commun. 2026 May 28;17:4771. doi: 10.1038/s41467-026-72123-2 (PMC13219400; doi:10.1038/s41467-026-72123-2)
Supplement: Supplementary file 1 — Supplementary Information File [file 41467_2026_72123_MOESM1_ESM.pdf]

Supplementary Information for

Direction-Specific Enhanced Diffusion of CO_2 in Chiral Hexagonal Boron Nitride Nanotubes

Manh-Thuong Nguyen,[a*] David J. Heldebrant,[a,b*], Jian Liu[a] and Abhoyjit Bhown,[c] Zhijie Xu[a]

[a] Pacific Northwest National Laboratory, Richland, Washington 99352, United States

[b] Washington State University, Pullman, Washington 99164, United States

[c] Electric Power Research Institute, Palo Alto, California 94304, United States

*Email: manhthuong.nguyen@pnnl.gov, david.heldebrant@pnnl.gov

S1. Methods

S1.1. Systems

We considered the h-BN nanotubes (BNNTs) of indices (5,5), (6,4), (7,3), (9,0), and (7,4), as generated using the TubeGen server. [1]

For binding energy screening, we used nanotubes/surfaces of which the chiral index and unit cell shown in Figure S1

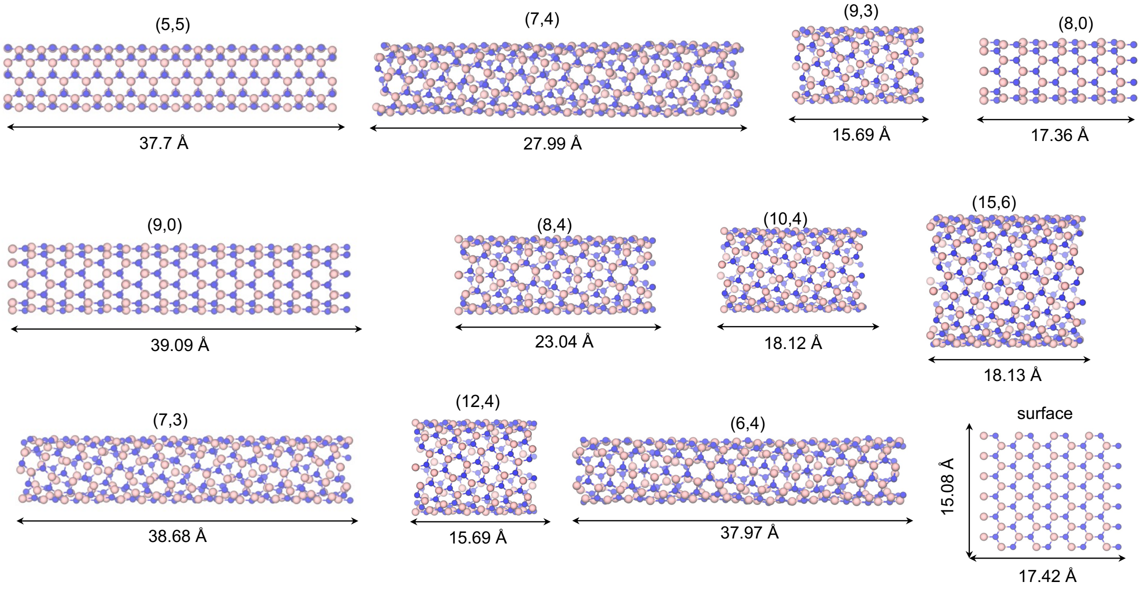

Figure S1. *The unit cell size and chiral index of hBNNTs used for binding energy calculations. Colour code: B in pink and N in blue.*

S12. Adsorption free energy

At 1 atm, the free energy of X in hBNNTs (X= CO2, N2) was approximated as[2]

$$\Delta G^0(X) \approx \Delta E(X) - \tilde{\mu}(\mathrm{T}, \mathrm{p}^0, \mathrm{X}),$$

in which ΔE is the calculated adsorption energy, and $\tilde{\mu}(\mathrm{T}, \mathrm{p}^0, \mathrm{X})$ is the chemical potential of molecule X at temperature T (T= room temperature in this study) and pressure p^0 = 1 atm, as taken from NIST-JANAF Thermochemical Tables.[3]

S1.3. Electrostatic potentials

Suppose that z is the tube axis direction, we calculated the xy-plane electrostatic potential for all points within a cylinder of radius R_c centered at the tube axis, Scheme S1, as

$$V(z) = \frac{1}{\pi R_c^2} \int_{x^2+y^2=0}^{x^2+y^2=R_c^2} V(x,y,z)dxdy$$

where V(x,y,z) is the electrostatic potential (calculated using DFT) at point (x,y,z). V(z) shown in Figure 3, main text, corresponds to R_c=3.0 Å.

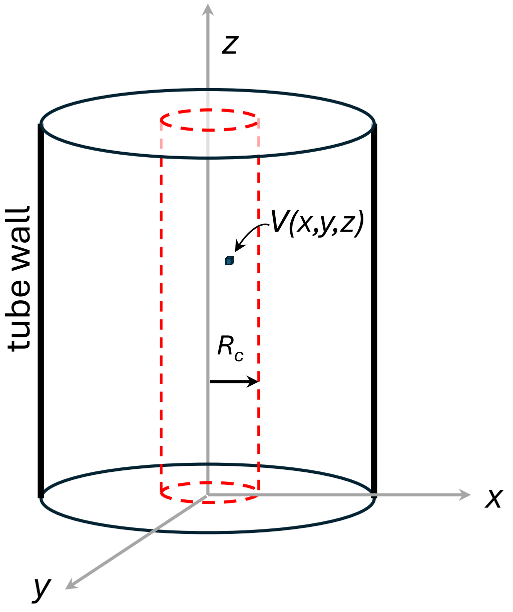

Figure S2. *Points within the cylinder (dashed lines) of the radius R_c centered at the tube axis used to calculate V(z)*

S1.4. Twist angle of hBNNTs

The twist angle θ for (n,m) hBNNTs relative to the armchair nanotube was calculated as[4]

$$\theta = \frac{\pi}{6} - \sin^{-1}\left(\frac{m\sqrt{3}}{2\sqrt{n^2+mn+m^2}}\right)$$

S1.5. Rotational frequency and torque calculations

The rotational frequency the OO axis about the tube direction ($\vec{z}$) was calculated as

$$f_p = |\frac{1}{2\pi}\omega_z| = |\frac{1}{2\pi}\frac{(\mathbf{r}\times\mathbf{v})_z}{|\mathbf{r}|^2}|$$

in which the coordinate

$$\mathbf{r} = \mathbf{r}_\mathrm{o} - \mathbf{r}_\mathrm{M}$$

and velocity

$$\mathbf{v} = \mathbf{v}_\mathrm{o} - \mathbf{v}_\mathrm{M}$$

with O being an oxygen atom of CO_2 and M is the midpoint of OO.

For the rotation of CO_2 (more precisely, rotation of C atom) about its OO axis, we adopted the following. Since the CO2 is flexible, it is necessary to separate its rotation and vibration. Here we used the approach introduced by Eckart.[5, 6] Very briefly, for each molecular dynamics frame, the coordinates and velocities of 3 CO_2 atoms are subtracted by that of CO_2's center of mass, the first frame was chosen as the reference. The Eckart alignment was then performed to determine rotation matrices, $\boldsymbol{R}$=$\boldsymbol{R}(t)$, from which the body-frame angular velocity tensor, $\boldsymbol{\Omega}(t) = \boldsymbol{R}^{\boldsymbol{T}}\dot{\boldsymbol{R}}$, was calculated. This allowed to determine body-frame angular velocity vector $\boldsymbol{\omega}(t)$. The rotational frequency of CO_2 about its OO axis was finally calculated by projecting $\boldsymbol{\omega}(t)$ onto the OO direction of the reference frame, with the $1/2\pi$ factor included.

The torque τ of a rigid, bent CO2 was calculated using the Euler's equations:

$$\tau = \boldsymbol{I}\dot{\boldsymbol{\omega}} + \boldsymbol{\omega} \times (\boldsymbol{I}\boldsymbol{\omega})$$

where $\boldsymbol{I}$ is the inertia tensor of the molecule.

S2. Results

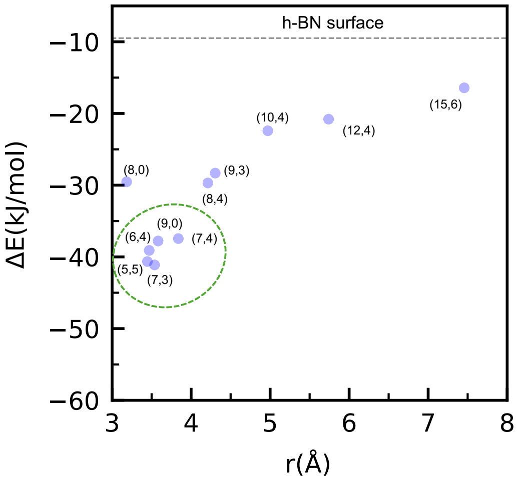

Figure S3. *Binding energy of N_2 in hBNNTs. The dashed oval indicates five systems with highest binding energies.*

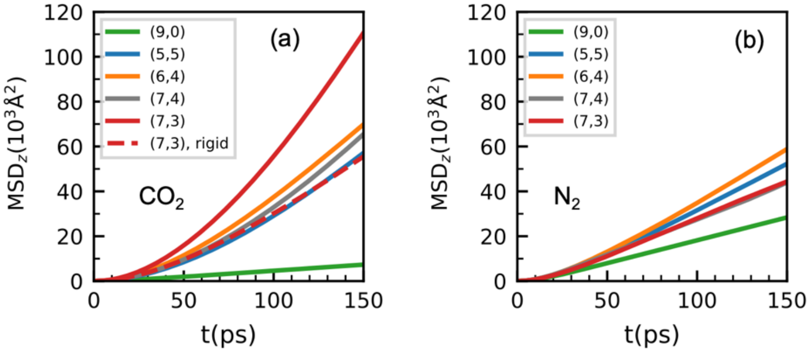

Figure S4. *MSD_z of CO_2 (a) and N_2 (b) in hBNNTs from MLIPMD. The diffusion coefficient is calculated as the slope of the linear part from 100 to 150 ps.*

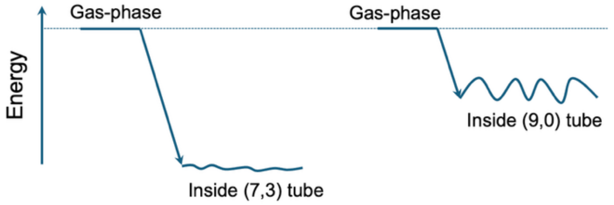

Figure S5. *Schematic illustration of the energy profile as gas-phase CO_2 enters and diffuses within hBNNTs. CO_2 exhibits stronger adsorption and a flatter energy landscape during diffusion in (7,3), whereas it experiences weaker adsorption and a rougher energy surface in (9,0).*

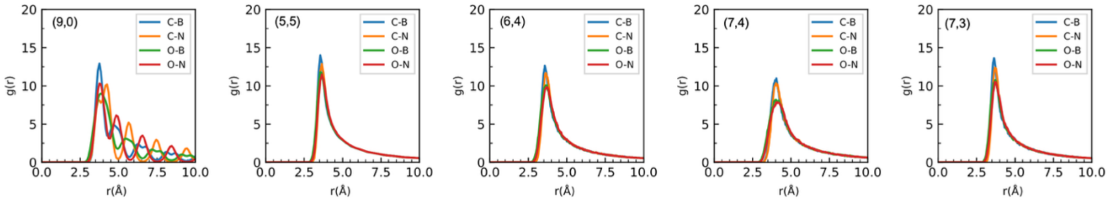

Figure S6*. Radial distribution functions, g(r), of atom pairs, in which C and O are CO_2 atoms and B and N are hBNNT atoms.*

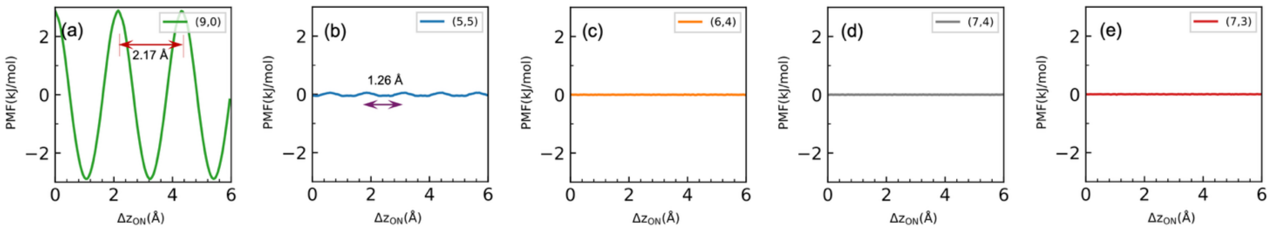

Figure S7. *The potential of mean force PMF=$-k_BT\ln[P(z_{ON})]$ with the collective variable being z_{ON} - the z-component of the O-N distance (with O being oxygen of CO2 and N being nitrogen of the tube).*

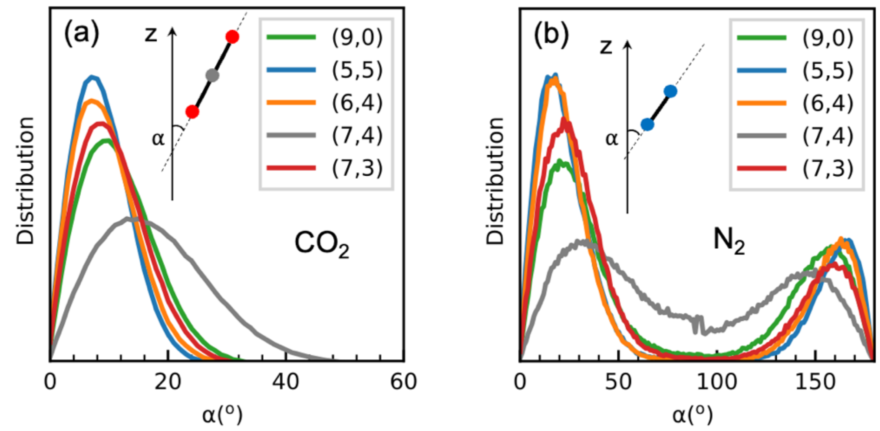

Figure S8.*(a) Distribution of the angle α between the CO_2 molecule (OO direction) and the tube direction (z). (b) Distribution of the angle α between the N_2 axis and the tube direction. Finite values in the 50°-130° range indicate that N_2 tumbles during diffusion.*

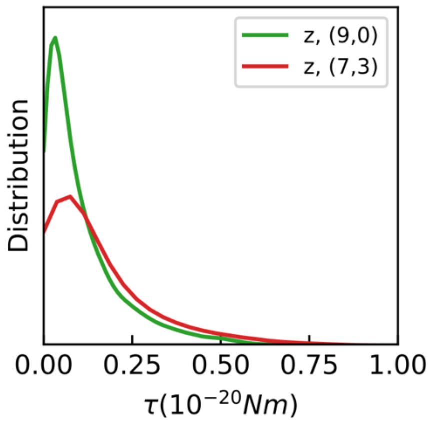

Figure S9. *The z-component the torque of a rigid, bent CO2 (with the C-O bond fixed at 1.175 Å and the O-C-O angle fixed at 174.95^o).*

***Table S1**. Root mean squared error (RMSE) and mean absolute error (MAE) for the energy E (per atom, 10^{-5} eV) and the force F (10^{-3} eV/ Å) of the training/validation/test sets.*

hBNNT	CO_2				N_2			
	E		F		E		F	
	RMSE	MAE	RMSE	MAE	RMSE	MAE	RMSE	MAE
(9,0)	5/5/5	4/4/4	24/24/24	18/18/18	6/6/6	5/5/5	25/25/25	20/20/20
(5,5)	4/4/4	3/3/3	24/24/24	18/18/18	7/7/7	6/6/6	24/24/24	19/19/19
(6,4)	4/4/4	3/4/4	25/25/25	18/19/19	6/6/6	5/5/5	25/25/25	19/19/19
(7,4)	5/5/5	5/5/5	27/27/27	21/21/21	5/5/5	4/4/4	26/26/26	20/20/20
(7,3)	7/7/7	7/7/7	24/24/24	18/19/19	5/5/5	4/4/4	25/25/25	19/20/20
$(7,3)_{\text{rigid mol}}$	4/4/4	3/3/3	24/24/24	19/19/19				

References

1. J. T. Frey and D. J. Doren Tw-i, http://turin.nss.udel.edu/research/tubegenonline.html), University of Delaware, Newark DE, 2011. TubeGen 3.4 (web-interface, http://turin.nss.udel.edu/research/tubegenonline.html), J. T. Frey and D. J. Doren, University of Delaware, Newark DE, 2011.).

2. Reuter K, Scheffler M. Composition, structure, and stability of ${\mathrm{RuO}}_{2}(110)$ as a function of oxygen pressure. *Phys Rev B* **65**, 035406 (2001).

3. NIST. *NIST-JANAF Thermochemical Tables* (2024).

4. Nishihara T, Takakura A, Matsui K, Itami K, Miyauchi Y. Statistical verification of anomaly in chiral angle distribution of air-suspended carbon nanotubes. *Nano Lett* **22**, 5818-5824 (2022).

5. Eckart C. Some studies concerning rotating axes and polyatomic molecules. *Physical Review* **47**, 552 (1935).

6. Wilson EB, Decius JC, Cross PC. *Molecular vibrations: the theory of infrared and Raman vibrational spectra*. Courier Corporation (1980).
