# Supplementary material for: Direction-specific enhanced diffusion of CO2 in chiral hexagonal boron nitride nanotubes
Source: Nat Commun. 2026 May 28;17:4771. doi: 10.1038/s41467-026-72123-2 (PMC13219400; doi:10.1038/s41467-026-72123-2)
Supplement: Supplementary file 3 — Description of Additional Supplementary Files [file 41467_2026_72123_MOESM3_ESM.pdf]

Description of Additional Supplementary Files

Supplementary Code 1

Description: DeePMD input file for training Machine Learning interatomic potentials.

Supplementary Movie 1

Description: Side view of a 7,3 chiral BN tube with a CO_2 molecule precessing while diffusing left to right inside. Boron atoms are pink, Nitrogen atoms are blue. Movie represents 2 picoseconds of time lapse.

Orange bar: a vector confined to the COO plane and orthogonal to the O-O axis

Green bar: vector aligned with the O-O axis

Supplementary Movie 2

Description: Top view of a 7,3 chiral BN tube with a CO_2 molecule precessing counterclockwise while diffusing inside. Boron atoms are pink, Nitrogen atoms are blue. Movie represents 2 picoseconds of time lapse.

Orange bar: a vector confined to the COO plane and orthogonal to the O-O axis

Green bar: vector aligned with the O-O axis

Supplementary Movie 3

Description: Side view of a 9,0 chiral BN tube with a CO_2 molecule precessing clockwise and counterclockwise while diffusing back and forth inside. Boron atoms are pink, Nitrogen atoms are blue. Movie represents 2 picoseconds of time lapse.

Orange bar: a vector confined to the COO plane and orthogonal to the O-O axis

Green bar: vector aligned with the O-O axis

Supplementary Movie 4

Description: Side view of a 9,0 chiral BN tube with a CO_2 molecule precessing clockwise and counterclockwise while diffusing. Boron atoms are pink, Nitrogen atoms are blue. Movie represents 2 picoseconds of time lapse.

Orange bar: a vector confined to the COO plane and orthogonal to the O-O axis

Green bar: vector aligned with the O-O axis
